# Effect of prenatal different auditory environment on learning ability and fearfulness in chicks

**DOI:** 10.5713/ab.21.0470

**Published:** 2022-01-05

**Authors:** Shuai Zhao, Chunzhu Xu, Runxiang Zhang, Xiang Li, Jianhong Li, Jun Bao

**Affiliations:** 1 College of Animal Science and Technology, Northeast Agricultural University, Harbin 150030, Heilongjiang, China; 2 College of life Science, Northeast Agricultural University, Harbin 150030, Heilongjiang, China; 3 Key Laboratory of Chicken Genetics and Breeding, Ministry of Agriculture and Rural Affairs, Harbin 150030, China

**Keywords:** Auditory Stimulation, Chicks, Fearfulness, Learning Ability

## Abstract

**Objective:**

Early environmental enrichment in life can improve cognition in animals. The effect of prenatal auditory stimulation on learning ability and fear level in chick embryos remained unexplored. Therefore, this study investigated the effect of prenatal auditory stimulation on the learning ability and fear level of chicks.

**Methods:**

A total of 450 fertilized eggs were randomly divided into 5 groups, including control group (C), low-sound intensity music group (LM), low-sound intensity noise group (LN), high-sound intensity noise group (HN) and high-sound intensity music group (HM). From the 10th day of embryonic development until hatching, group LM and group LN received 65 to 75 dB of music and noise stimulation. Group HN and group HM received 85 to 95 dB of noise and music stimulation, and group C received no additional sound. At the end of incubation, the one-trial passive avoidance learning (PAL) task and tonic immobility (TI) tests were carried out, and the serum corticosterone (CORT) and serotonin (5-HT) concentrations were determined.

**Results:**

The results showed that compared with the group C, 65 to 75 dB of music and noise stimulation did not affect the PAL avoidance rate (p>0.05), duration of TI (p>0.05) and the concentration of CORT (p>0.05) and 5-HT (p>0.05) in chicks. However, 85 to 95 dB of music and noise stimulation could reduce duration of TI (p<0.05) and the concentration of CORT (p<0.05), but no significant effect was observed on the concentration of 5-HT (p>0.05) and PAL avoidance rate (p>0.05).

**Conclusion:**

Therefore, the prenatal auditory stimulation of 85 to 95 dB can effectively reduce the fear level of chicks while it does not affect the learning ability.

## INTRODUCTION

Environmental enrichment may enhance the brain's normal development and the plasticity of brain structure and function and improve cognitive ability [1]. High cognitive ability can help animals to adapt to the changed social environment. Environmental enrichment is known to reduce the fearfulness of animals. For instance, chicks provide various novel stimuli, such as balls, buttons, thimbles, which can reduce fear in chicks. Fearfulness is an evolutionarily adaptive ability against danger [2]. Exaggerated fear responses may cause smothering and mortality in animals, which is a general concern in the poultry industry.

Prenatal environmental enrichment affects brain development and behavioral expression in animals [3]. For example, light [4] and sound [5] showed important prenatal experience that affects chick’s behavior. In particular, prenatal auditory stimulation can result in the early maturation of the auditory system [6] and improve the learning ability of newborn animals [7]. Studies have shown that prenatal exposure to the sound results in increased neurogenesis in the hippocampus and enhance the spatial learning ability of rats [8] and chicks [3]. Thus, prenatal auditory stimulation plays a vital role in the development of neonates.

In auditory stimuli, music has a high functional value. Music exposure was considered an effective way to enrich the auditory system. As a result, music has been increasingly used as a form of acoustic stimulation to improve fowls' neurological development and welfare during incubation. For example, 65 dB and 110 dB of music exposure in the prenatal period can improve spatial learning ability by increasing the expression of hippocampal synaptic protein [6] and brain-derived neurotrophic factor (BDNF) [5]. In artificial incubation, there are many technologies, such as ventilation, egg flipping, humidity and refrigeration systems used to ensure optimal conditions for the development of poultry embryos. During this process, noise caused by the engine and fan was inevitable, which results in a sound intensity approach up to 95 dB [9]. During the incubation period, 110 dB noise exposure can affect the development of the poultry brain, reduce brain volume and weight [10], increase thyroxine, and impair learning and memory ability [11]. In other studies, the noise of commercial hatcheries at 90 dB was beneficial to artificial hatching, led to earlier hatching, higher hatchability, better chick quality [12]. Furthermore, there is evidence that the species-specific calls can also accelerate the hatching process and improving the hatching quality, which was beneficial to the anti-stress response and spatial orientation ability of day-old chicks [12,13].

There are several studies on the effects of sound exposure on neurodevelopmental and incubation responses to the animals. A few studies were focused on the effect of prenatal sound exposure on fearfulness and the learning ability of newborn animals. Being a precocial species, chicks will exhibit responses to air borne sound around embryonic days 11/12 [14]. Meanwhile, they have strong learning ability and memory on the first day after incubation, enabling them to complete various cognitive tasks and show a preference for sound [15]. Thus, we choose chicks as the experimental model. The aim of this study determines whether the addition of auditory stimuli in the prenatal of poultries could reduce fear and improve cognitive ability by detection of the one-trial passive avoidance learning (PAL) task, tonic immobility (TI) test and the concentrations of serotonin and corticosterone in neonatal chicks.

## MATERIALS AND METHODS

### Subjects and general procedures

All experiments were approved by and conducted according to the guidelines of the Institutional Animal Care and Use Committee of Northeast Agriculture University (Ethic code: IACUCNEAU20190717).

Roman-white domestic chicks (*Gallus gallus domesticus*) were used as the experimental model. Fertilized eggs (day 0) of healthy laying hens (weigh of 60±5 g) were obtained from a local registered commercial rearing unit and incubated in an incubator (Incubator-LN2-S, Dezhou Limin Livestock Machinery Co., Ltd., Shandong, China). Incubation conditions were maintained at 50% to 70% humidity and temperature of 37°C (±1°C). The levels of temperature and humidity, and air quality in the incubator were controlled. The lighting schedule was 12 L:12 D, and eggs automatically turned on every 2 h throughout the incubation period (21 d), which was done to prevent adhesions of embryos. The development of the embryo can be exposed to the egg lamp on the 8th day, in which we examined all eggs and removed the unfertilized eggs.

### Experimental groups and auditory stimulation

A total of 450 fertilized eggs were divided into the following five groups (n = 90 for each group) and placed in five separate incubations:

Group C: Control group; the eggs were incubated under normal conditions with no additional sound exposure.
Group LM: Low-intensity music group; the eggs were exposed to Mozart’s classical music (Mozart’s String Quartets, K428, K525, K458) stimulus of intensity ranged from 65 to 75 dB from 10th day until hatching.
Group LN: Low-intensity noise group; the eggs were exposed to the noise stimulus of intensity ranged from 65 to 75 dB from 10th day until hatching. The noise was the pre-recorded sound of ventilation fans and the commercial incubator.
Group HN: High-intensity noise group; the eggs were exposed to the noise stimulus of intensity ranged from 85 to 95 dB from 10th day until hatching. The noise was the pre-recorded sound of ventilation fans and the commercial incubator.
Group HM: High-intensity music group; the eggs were exposed to Mozart’s classical music (Mozart’s String Quartets, K428, K525, K458) stimulus of intensity ranged from 85 to 95 dB from 10th day until hatching.

During this period, experimental groups were exposed to sounds every day from 7:00 to 19:00. During this period, the sounds were played (on for an hour/off for an hour) in loops through two built-in speakers (SPA311; Philips (China) Investment Co., Ltd., Shanghai, China) connected to a laptop computer provided with an automatic setting. The intensity of the sounds emitted by the speakers was calibrated from measurements taken by a decibel meter (VICTORVC824; Shenzhen city station win Technology Co. Ltd., Guangdong, China) at the center of the incubation chamber (24 cms apart).

All incubators produced about 45 dB sound intensity. However, the sound intensity was so far below than that of the experimental group. So, the possibility of acoustic interference was excluded.

### The one-trial passive avoidance learning task

The one-trial PAL task exploits the spontaneous tendency of chicks to peck at objects for examining learning and memory in the young chicks [16].

After hatching, the chicks of five groups were maintained in five cartons (38×31×25 cm) and located in a temperature-controlled room (32°C to 35°C). *Ad libitum* water was given, but the chicks were fasted. On the second day, 24 chicks (male: female = 1:1) from each treatment group were randomly selected and transferred to cartons (20×20×25 cm) in randomly assigned pairs, and each chick was illuminated with a 25 W LED light. Before pre-training, they were familiarized with the experimental environment at 30°C to 32°C for at least 30 min. In each pair, one chick was marked by spraying black color on its head for identification during the data recording.

At pre-training, the chicks were presented with a water-dipped white bead (2 mm diameter) for 10 s to encourage the chicks’ natural pecking response. There was 5 min difference between each trial in this procedure, and three pre-training trials were performed. Each chick was presented with a red bead (4 mm diameter) dipped with 99% methyl anthranilate (MeA) for 10 s after 25 min of pre-training trial. MeA is a substance of aversive taste, and chickens typically respond to it with a disgust response, such as shaking their heads, closing their eyes, and occasionally wiping their beaks on the floor of the box. Test of memory was conducted at 120 min after training. The chicks were presented first with a dry red bead (identical to the one in the training trial), and then a dry white bead (identical to the one in the pretraining trial) on two trials (10 s), 5 min interval. In order to exclude the influence of order and color, the first 6 pairs of chicks were presented with red beads, followed by white beads, and the last 6 pairs were presented with white beads and then red beads. Each chick's behavior was recorded as pecking or not pecking.

The memory retention was calculated as a percent avoidance score (the number of chicks in each group that avoids the red bead but pecks the white on test ×100%/total number of the trained chicks) [17]. Each chick was trained and tested only once. During the PAL task, the chicks which did not peck during at least one of the three pre-training trials, were excluded from the analysis in the training trial or the second testing trials.

### Tonic immobility

On the second day of hatching, 20 chicks (male:female = 1:1) were randomly collected from each group and were tested individually for the duration of TI in a separate room. The chicks which had previously been tested in the PAL task and identified due to the black spray paint on its head, were not selected for the TI test. The TI test was carried out according to the method described previously [18]. Briefly, the individual chick was placed on its back in a wooden, U-shaped cradle. The chick was then restrained with one hand on its sternum while holding the head and neck with the other hand to induce TI. The induction time was 15 s. The experimenter slowly released his hands, and the duration of TI reaction, that was, the freeze, was recorded. Freezing was defined as the absence of movement, except for any movement related to respiration and tremors. At the end of the induction period, hand pressure was gradually lifted so that if the chick could still move. Another induction period was started immediately, until the movement ceased. After removal of the hands, a stopwatch was started. The experimenter was then retreated, and the behavior of the chick was observed. If the chick righted itself in less than 10 s, it was considered that TI had not been induced, and the restraint procedure was repeated (maximum three times). If a chick did not show a righting response over the 15 min test period, a maximum score of 900 s was given for duration.

### Sample collection and estimation of blood corticosterone and serotonin concentration

Tissue samples were collected after the TI and PAL tasks on the second day of hatching. Eight chicks (male:female = 1:1) were randomly selected from each group and decapitated immediately for tissue collection. The time between catching and tissue sampling was less than 2 min per chick. Blood was collected into 1.5 mL Eppendorf (EP) tubes. The blood was clotted for 24 h at 4°C, and the EP tubes were spun down in an ALLSHENG iCen24 centrifuge (Hangzhou Allsheng Instruments Co., Ltd., Hangzhou, China) for 15 min at 4,000 rpm to separate the clot. The serum samples were poured off into 1.5 mL EP tubes and stored at −80°C until further analysis. The concentration of serum corticosterone (CORT) and serotonin (5-HT) was measured using a commercially available ELISA kit (Nanjing Jiancheng Bioengineering Institute, Nanjing, China) and the corresponding procedures. The intra-assay coefficient of variation of the kit was less than 12%, and the assay ranged from l0.2 to 60 ng/mL.

### Statistical analysis

Statistical analyses were performed using SPSS 21 for Windows (SPSS Inc., Chicago, IL, USA). All data were tested for normal distribution using the Kolmogorov–Smirnov test. Intergroup differences at the TI duration, CORT and 5-HT concentrations, were analyzed by unpaired Student's t-test. Fisher's Exact Test was conducted on memory retention. The results were expressed as mean±standard error of the mean, and differences were considered statistically significant at p<0.05.

## RESULTS

### Effect of prenatal sound exposure for PAL task in chicks

The avoidance ratio for chicks from the control (C), low-intensity music (LM), low-intensity noise (LN), high-intensity noise (HN) and high-intensity music (HM) groups were presented in (Table 1). There were no significant differences in avoidance ratio among the groups (LM, p = 1), (LN, p = 1), (HN, p = 0.422), and (HM, p = 0.214) compared to group C. In total, 22.5% of the chicks did not meet the inclusion criteria and were excluded from the analysis.

### Effect of prenatal sound exposure for tonic immobility in chicks

**Figure 1:**
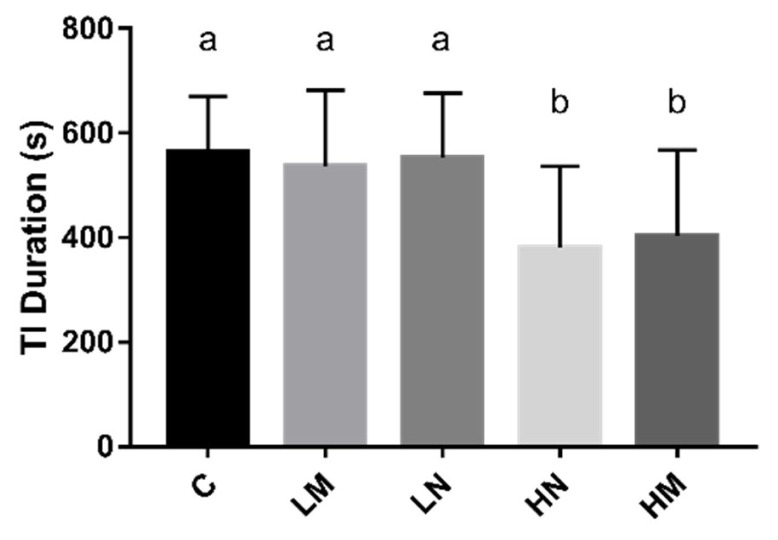
The effect of prenatal sounds exposure on TI duration (s) in chicks. n = 20/treatment. TI, tonic immobility; ANOVA, analysis of variance. ^a,b^ Means with different superscripts represents significant differences between groups (p<0.05), the same superscripts represent no significant differences (p>0.05).

Mean values for the duration of tonic immobility per treatment were presented in (Figure 1). The group HM (p<0.05) and group HN (p<0.05) displayed significantly shorter duration of tonic immobility compared to chicks from group C. There was no significant difference in TI duration in group LM (p>0.05) and group LN (p>0.05) compared to group C.

### Effect of prenatal sound exposure for levels of serum corticosterone and serotonin in chicks

**Figure 2:**
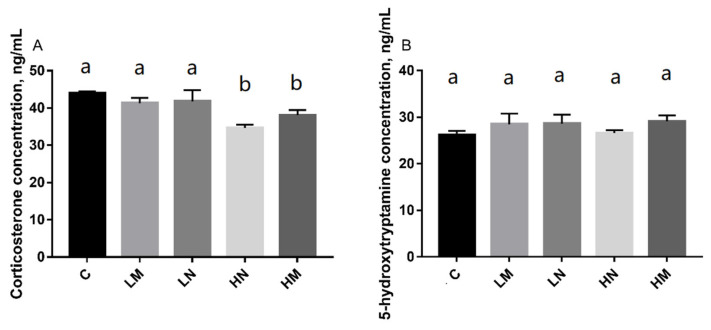
The effect of prenatal auditory exposure on CORT and 5-HT concentration (ng/mL) in chicks. n = 8/treatment. ANOVA, analysis of variance. ^a,b^ Means with different superscripts represents significant differences between groups (p<0.05), the same superscripts represent no significant differences (p>0.05). CORT, serum corticosterone; 5-HT, serotonin.

The CORT concentration (ng/mL, mean±standard deviation [SD]) of chicks in C, LM, LN, HN, and HM groups was presented in (Figure 2). This study showed that the chicks in group HN (p<0.05) and group HM (p<0.05) had significantly lower serum corticosterone concentrations than group C, but there were no significant differences among the LM (p>0.05), LN (p>0.05) and C groups.

The serum serotonin (5-HT) concentration (ng/mL, mean ±SD) of chicks in the C, LM, LN, HN, and HM groups was presented. There were no significant differences in 5-HT concentration among the groups LM (p>0.05), LN (p>0.05), HN (p>0.05), and HM (p>0.05) compared to group C.

## DISCUSSION

The majority of the chicken auditory system development occurs in ovo and matures earlier than other sensory systems [19]. Therefore, sound exposure of prenatal, as an environmental enrichment method, provides an environmental stimulus for developing the avian sensory system. In this study, there were no differences in the PAL avoidance rate, duration of TI, CORT, and 5-HT concentrations of chicks between group C, group LM, and group LN. However, those of group HM and group HN were significantly difference than groups C, LN, and LM, which may be due to the acoustic insulation effect of eggshells on sound. Intact eggshells presented an insulation value of 17.14% at 70 dB that measured at the egg chamber with a special microphone [9]. This results in sound intensity after passing through the eggshell would not be enough to elicit a response from the embryo at 65 to 75 dB. Tong et al [20] found that exposure to 72 dB did not affect the growth, hatchability, mortality and hormone level of the chicken embryos, which was also consistent with our results. Therefore, no significant effect was observed on chicken embryos exposure to 65 to 75 dB music and noise.

Environmental factors can influence the development of the brain throughout embryonic developmental stage in humans and other animals. Prenatal music stimulation promotes the expression of hippocampal synaptogenesis proteins and BDNF, enhancing spatial orientation [7] and cognitive abilities [21] of chicks, even when the sound intensity of music reaches 110 dB [6]. In this study, exposed to 85 to 95 dB music, the chicks did not show a higher avoidance rate in the PAL task. This may be because the sound intensity was not strong enough to elicit a response from the embryo in learning ability and memory. Interestingly, in this study found that prenatal exposure to 85 to 95 dB noise made the same effect in the PAL task.

Fear response of poultry has been increasingly used to evaluate the welfare status of poultry [22]. The duration of TI is a well-validated method to evaluate the fear level of poultry [23]. The longer the duration of immobility of poultry, the stronger the fear [22]. Environmental enrichment effectively decreases stress and fearfulness and improves the physical and psychological well-being of hens [24]. Sound (rhythmic and patterned) stimulus as an environmental enrichment method during hatching, it can reduce the fear level of chicks after hatching [6,19]. In this study, there were a significantly shorter TI duration of prenatal exposure to 85 to 95 dB music and noise environment was observed compared with the control group in day-old chicks, which suggests that the prenatal exposure to 85 to 95 dB music and noise could reduce the fear level.

In this study, there was no significant difference in the concentration of 5-HT between the treatment groups. However, the serum CORT concentration of chicks under prenatal exposure of 85 to 95 dB music and noise was significantly lower than that in the control group. CORT and 5-HT are the indicators of various forms of stress in fowls [25]. The concentrations of CORT in the blood were associated with thirst, hunger, heat, fear, stress or the barren environment in chicks [26]. The lower CORT concentration indicates a lower stress level. This is consistent with the decrease of CORT concentration caused by 85 to 95 dB music and noise stimuli in this study. Fear has been shown to cause changes in 5-HT concentrations [27]. However, in this study, the distinction of the degree of fear was not reflected accurately in the concentration of 5-HT. This may be because a large amount of 5-HT in the blood is mainly carried by platelets [28], and serum 5-HT concentration is not the best choice. In future studies, whole blood 5-HT concentration should be selected to provide a basis for physiological measurement [29].

In summary, whether the effects of auditory stimulation during egg incubation are beneficial or detrimental well depend on rhythmicity and timing of exposure [12,14,27,28]. In this study, the music was the complex rhythmic sound, and the noise was the mixed noise of fans and machinery in the incubator, which was patterned sound. At a higher intensity, these patterned and rhythmic sounds have no harmful effects on the embryonic development of the auditory system, and spatial orientation and the ability to resist stress [3,28–30]. However, in other studies, unpredictable noise, such as vehicle honking, was used [6,30,32]. These dissonant and noisy structures are associated with aggression, fear and defense [27,28], which may be the main reason for the different results. In addition, the study of Hedlund et al [33] found that chicks have elevated levels of CORT hatched in commercial hatchery (the noise was ventilation fans at 90 dB) compared to chicks hatched under calm conditions. It is contrary to our results, which may be because the commercial hatchery noise caused by fans was continuous for 24 h/d during the incubation period, while the sound stimulation used in this study is intermittent for 6 h/d, which was far less than the time of exposure in commercial hatchery. This also shows that long-term continuous sound exposure before hatching is harmful to the development of embryonic, while intermittent short-term sound stimulation can have a beneficial effect. Further research should explore the effects of different timing of sound exposure in incubation period for newborn chicks.

## CONCLUSION

This study indicated that prenatal exposure to 65 to 75 dB music and patterned noise could not produce effective stimulation for embryonic development. However, beneficial effects on embryonic development for chicks prenatally exposed to music and patterned noise were observed at 85 to 95 dB (6 h per day, on for an hour/off for an hour), reducing the fear level and serum CORT concentration, but there was no effect on the learning ability. Our study suggests that prenatal 85 to 95 dB music and patterned noise stimulation can positively reduce fearfulness in chicks.

## Figures and Tables

**Table 1:** The effects of prenatal auditory exposure on learning in day-old chicks

Treatment^1)^	N	Avoidance (%)	p-value
C	16	68.75	-
LM	20	65	1
LN	19	68.42	1
HN	20	85	0.422
HM	18	88.89	0.214

Results presented as percent avoidance for the passive avoidance (= the number of chicks in each group that avoided the red bead but pecked the white on test ×100% /total number of the trained chicks) learning tasks at 120 min after training. Numbers of chicks in each group are presented in the N.

Statistical difference is represented as p by Fisher's Exact Test compared to group C.

1) C, control group; LM, low-sound intensity music group; LN, low-sound intensity noise group; HN, high-sound intensity noise group; HM, high-sound intensity music group.
